# Delivering a Group-Based Quality of Life Intervention to Young Adult Cancer Survivors via a Web Platform: Feasibility Trial

**DOI:** 10.2196/58014

**Published:** 2024-12-04

**Authors:** Rina S Fox, Tara K Torres, Terry A Badger, Emmanuel Katsanis, DerShung Yang, Stacy D Sanford, David E Victorson, Betina Yanez, Frank J Penedo, Michael H Antoni, Laura B Oswald

**Affiliations:** 1 Division of Advanced Nursing Practice and Science, University of Arizona College of Nursing, Tucson, AZ, United States; 2 University of Arizona Cancer Center, Tucson, AZ, United States; 3 Department of Psychology, University of Arizona, Tucson, AZ, United States; 4 Department of Pediatrics, University of Arizona College of Medicine - Tucson, Tucson, AZ, United States; 5 BrightOutcome, Inc., Buffalo Grove, IL, United States; 6 Department of Medical Social Sciences, Northwestern University Feinberg School of Medicine, Chicago, IL, United States; 7 Robert H. Lurie Comprehensive Cancer Center of Northwestern University, Chicago, IL, United States; 8 Department of Psychology, University of Miami, Miami, FL, United States; 9 Cancer Control Research Program, Sylvester Comprehensive Cancer Center, Miami, FL, United States; 10 Department of Health Outcomes and Behavior, Moffitt Cancer Center, Tampa, FL, United States

**Keywords:** cancer survivors, survivorship, clinical trials, psychosocial intervention, usability testing, digital therapeutics, young adults, nonrandomized

## Abstract

**Background:**

Young adult (YA) cancer survivors frequently report unmet health information and peer support needs, as well as poor health-related quality of life (HRQOL). YAs also have expressed a desire that behavioral interventions be convenient. In response to this, our team has developed a 10-week, group-based, supportive care intervention titled TOGETHER to improve YA cancer survivors’ HRQOL. TOGETHER is delivered via videoconference and has shown initial feasibility, acceptability, and promise for improving HRQOL among YA survivors.

**Objective:**

In an effort to increase convenience, the goal of this 2-part study was to design and test a website to host the TOGETHER intervention for YA cancer survivors aged 18‐39 years at the time of participation and aged 15‐39 years at the time of initial cancer diagnosis.

**Methods:**

In part 1, we leveraged an existing web-based platform and adapted it to meet the needs of TOGETHER. We conducted 3 iterative waves of usability testing with 3 YAs per wave to refine the website. In part 2, we conducted a single-group feasibility trial of TOGETHER using the website. Primary outcomes were feasibility (ie, recruitment, retention, and attendance) and acceptability (ie, satisfaction).

**Results:**

Usability testing participants (n=9) indicated that the TOGETHER website was easy to use (mean 5.9, SD 1.3) and easy to learn (mean 6.5, SD 0.9; possible ranges 1‐7). Qualitative feedback identified needed revisions to the aesthetics (eg, images), content (eg, session titles), function (eg, clarity of functionality), and structure (eg, expandable sections), which were implemented. In the feasibility trial, participants (n=7) were an average of 25 (SD 4.7) years old and mostly non-Hispanic White (n=4, 57%). Recruitment (58%) and retention (71%) rates and average session attendance (mean 7.1 , SD 4.2) supported feasibility. Participant agreement with positive statements about TOGETHER and average satisfaction ratings (mean 5.06, SD 1.64; possible range: 1‐7) demonstrated acceptability.

**Conclusions:**

Results supported the usability, feasibility, and acceptability of the TOGETHER program and website. By providing the content digitally, the program effectively addresses YAs’ expressed preference for convenience. Future studies are needed to increase TOGETHER’s efficiency and explore its efficacy for improving targeted outcomes.

## Introduction

Young adult (YA) cancer survivors diagnosed between the ages of 15 and 39 years are a rapidly growing population [1]. YAs face unique challenges, such as cancer-related disruptions to reaching normative developmental milestones (eg, education, career, financial independence, emotional and sexual intimacy) [2,3] and biopsychosocial late effects of disease (eg, infertility) that may not be as salient for survivors in other age cohorts [2,4-6]. Perhaps as a result, YAs are at elevated risk for depression, anxiety, and stress [7-9] and frequently report lower levels of health-related quality of life (HRQOL) relative to both older and younger cancer survivors [6,10]. In addition to these challenges, most YA cancer survivors report unmet health information needs, and many report unmet peer support needs, which can further exacerbate low HRQOL [11,12]. Past research among older cancer populations aged>50 years has identified evidence-based approaches, such as interventions grounded in cognitive behavioral therapy, that improve HRQOL [13-16]. However, evidence-based strategies to improve HRQOL that meet the unique needs and preferences of YA cancer survivors are limited.

To address this need, our team developed a supportive care intervention specifically designed to improve YA cancer survivors’ HRQOL, called TOGETHER [17]. The TOGETHER content was derived and adapted for YAs from 2 supportive care interventions with established efficacy for improving HRQOL in other cancer survivor populations: Cognitive Behavioral Stress Management [18] and Health Education [19]. In addition to the strong support for their efficacy, these 2 interventions were selected, in part, because they can be remotely delivered via videoconference in a group setting, in accordance with YAs’ documented preferences that interventions should be convenient [20-22] and that peer support should be available [22]. Program content was adapted with iterative input from YA cancer survivors via focus groups. In subsequent preliminary testing, 2 intervention groups demonstrated that TOGETHER was feasible and acceptable [17].

Although the first iteration of TOGETHER was well received, it was available only as a static, noninteractive, PDF manual, which diminished the intervention’s convenience. In response to this and building on our foundational work, we conducted a 2-part study to design and test a website to host and deliver TOGETHER. In the first part of this study, we leveraged an existing digital platform that has historically been used to deliver similar supportive care interventions to other cancer survivor populations and adapted it to meet the needs of the TOGETHER program. Consistent with a rapid prototyping approach [23], we iteratively refined the platform based on feedback gathered from YA cancer survivors in 3 waves of usability testing. In the second part of the study, we tested the feasibility and acceptability of delivering TOGETHER via the adapted website in a single-arm, single-group feasibility trial. We hypothesized that TOGETHER would be feasible and acceptable based on predetermined benchmarks for recruitment, retention, average session attendance, and average participant satisfaction.

## Part 1: Building the TOGETHER Website

### Methods

#### Participants

Participants were YA cancer survivors aged 15‐39 years at the time of initial cancer diagnosis and aged 18‐39 years at the time of participation in this study. All participants had completed curative treatment at least 1 month but no more than 5 years before enrollment. Participants were also fluent in English, able to give informed consent, and not currently experiencing a psychiatric or neurological disorder that could impair their participation. YAs were recruited from the University of Arizona Comprehensive Cancer Center and community-based cancer advocacy groups in Tucson, Arizona.

#### Ethical Considerations

Study procedures were reviewed and approved by the University of Arizona Institutional Review Board (IRB #STUDY00000717). All participants provided written informed consent.

#### Procedures

To build the initial version of the TOGETHER website, we leveraged an existing digital platform developed by BrightOutcome Inc. [24]. The platform is a customizable, Health Insurance Portability and Accountability Act–compliant, mobile-friendly, password-protected website designed to facilitate remote delivery of course-based supportive care interventions and host live group videoconference sessions. We began by inputting the TOGETHER content into the platform’s infrastructure. Subsequently, we held 3 waves of usability testing with 3 participants per wave (n*=*9 total) based on prior research showing that 9 usability testers are needed to find moderately hard-to-find problems with 75% certainty [25].

Individual usability testing sessions were held in person and each lasted approximately 60 minutes. During these sessions, participants were introduced to a prototype of the TOGETHER website and asked to think aloud while completing a series of prescribed tasks (eg, log in, navigate from the home dashboard to session content). Participants were then asked to provide feedback on the appeal, clarity, comprehensibility, and aesthetic of the website. After each wave of testing, participant feedback was integrated into the website, and testers in the subsequent wave were shown the modified version. We iteratively incorporated stakeholder feedback in this way to maximize user engagement with TOGETHER [26]. Usability testing participants were compensated US $30 for their time.

#### Measures

Participants completed the 11-item Ease of Use subscale and 4-item Ease of Learning subscale from the Usefulness, Satisfaction, and Ease of Use (USE) questionnaire [27,28]. Items are rated on a 7-point scale ranging from 1 (*Strongly Disagree*) to 7 (*Strongly Agree*) and averaged to yield a total score. Higher scores indicate better usability. Usability testing participants also self-reported demographic and medical information.

#### Analysis

Audio recordings of the usability testing sessions were transcribed verbatim by a third-party service (GMR Transcription Services, Inc.). Each transcript was reviewed by at least 2 reviewers using an analytic approach similar to Gale and colleagues’ [29] rapid qualitative analytic method to identify actionable feedback. Reviewers identified suggested changes to the website and supporting quotes using an analysis template in Microsoft Excel. The audio recordings were revisited as needed for content and wording clarifications. After independent review, coders compared and discussed results to achieve consensus. This process was completed after each round of usability testing, and changes to the website were implemented rapidly in response to participant feedback. In addition, descriptive statistics were used to summarize the demographic and clinical characteristics of study participants and to describe website usability per the USE individual items and subscale scores.

### Results

#### Participant Characteristics

Information about usability testers’ demographic and medical characteristics can be found in Table 1. Participants were an average of 27 years old (range 23‐37) at the time of study participation and predominantly White (n=8, 89%), with two-thirds identifying as Hispanic or Latine (n=6, 67%). Slightly more than half were female, had completed some college or specialized training, and were working full-time (n=5, 56% each). The most common cancer diagnoses reported were breast cancer (n=3, 33%) and leukemia (n=2, 22%), and the average age at diagnosis was 24 (range 19‐34) years.

**Table 1.** Sample characteristics. Unless otherwise specified, all variables represent patient-reported information at the time of study participation. Six participants were included in both the usability testing and feasibility trial samples.

Variable	Statistic	
	Usability testing(n=9)	Feasibility trial(n=7)
Age at study participation in years, mean (range)	27.8 (23-37)	25.1 (18-33)
Age at diagnosis in years, mean (range)	24.7 (19-34)	22.0 (17-32)
Gender, n (%)		
Male	3 (33)	2 (29)
Female	5 (56)	5 (71)
Nonbinary	1 (11)	0 (0)
Race, n (%)		
White	8 (89)	7 (100)
Native American or Alaskan Native	1 (11)	0 (0)
Hispanic or Latine, n (%)	6 (67)	3 (43)
Education, n (%)		
Partial high school	0 (0)	1 (14)
High school graduate	1 (11)	1 (14)
Partial college or specialized training	5 (56)	3 (43)
College or university graduate	3 (33)	2 (29)
Relationship status, n (%)		
Never married	4 (44)	4 (57)
Married or partnered	4 (44)	3 (43)
Divorced	1 (11)	0 (0)
Employment, n (%)^a^		
Working full time	5 (56)	3 (43)
Working part time	0 (0)	2 (29)
Not employed	2 (22)	2 (29)
Student	1 (11)	0 (0)
Missing	1 (11)	0 (0)
Household income, n (%)		
<US $10,000	1 (11)	1 (14)
US $10,000-US $39,999	3 (33)	2 (29)
US $40,000-US $59,999	2 (22)	1 (14)
US $60,000-US $100,000	3 (33)	2 (29)
>US $100,000	0 (0)	1 (14)
Cancer type at diagnosis, n (%)		
Bone and soft tissue	1 (11)	2 (29)
Breast	3 (33)	2 (29)
Colorectal	1 (11)	0 (0)
Leukemia	2 (22)	1 (14)
Lymphoma	1 (11)	1 (14)
Thyroid and endocrine	1 (11)	1 (14)
Stage at diagnosis, n (%)		
I	3 (33)	2 (29)
II	0 (0)	0 (0)
III	2 (22)	2 (29)
IV	1 (11)	1 (14)
Unknown or not reported	3 (33)	2 (29)

a For employment, participants were instructed to select the response that they felt most closely aligned with their employment status at the time of study participation.

#### Usability: Quantitative Results

The frequency and means of item-level usability ratings for both USE subscales can be found in Table 2. Mean scores on the Ease of Use subscale of the USE questionnaire (mean 5.94, SD 1.27) and each of the 10 items contained therein (means ranged from 5.22 to 6.33) supported the website’s usability. Results for the Ease of Learning subscale demonstrated it was also easy to learn based on both the overall subscale score (mean 6.50, SD 0.89) and the 4 individual item scores (means ranged from 6.44 to 6.56).

**Table 2.** Usability ratings.

Item	Mean (SD)	Number of participants that endorsed each response						
		1 (Strongly Disagree)	2	3	4 (Neutral–Neither Agree nor Disagree)	5	6	7 (Strongly Agree)
Ease of use								
Easy	6.00 (1.32)	0	0	1	0	1	3	4
Simple	6.22 (1,30)	0	0	1	0	0	3	5
User-friendly	6.33 (1.00)	0	0	0	1	0	3	5
Fewest steps possible	5.67 (1.50)	0	1	0	0	1	5	2
Flexible	5.78 (1.30)	0	0	1	0	2	3	3
Effortless	6.00 (1.32)	0	0	1	0	1	3	4
Use without instructions	5.67 (1.94)	1	0	0	0	2	2	4
No inconsistencies	5.22 (2.05)	1	0	1	0	2	2	3
Users like	6.22 (1.30)	0	0	1	0	0	3	5
Recover mistakes	6.11 (1.27)	0	0	1	0	0	4	4
Use successfully	6.11 (1.69)	0	1	0	0	1	1	6
Ease of learning								
Learned quickly	6.44 (1.33)	0	0	1	0	0	1	7
Easily remember	6.56 (0.88)	0	0	0	0	2	0	7
Easy to learn to use	6.44 (1.01)	0	0	0	1	0	2	6
Quickly skillful	6.56 (0.73)	0	0	0	0	1	2	6

#### Usability: Qualitative Results

A screenshot of the TOGETHER website can be found in Multimedia Appendix 1. YA cancer survivors described the website as “clean,” “neat,” “visually pleasing,” “user-friendly,” “straightforward,” “organized,” “intuitive,” and “easy to navigate.” Participants particularly valued that there were multiple ways to access or complete various website functions. For example, participants expressed appreciation that program exercises and home practices could be completed digitally within the website or downloaded as a PDF to be completed offline. Participants identified several strengths related to the aesthetics, content, function, and structure of the website, as well as recommendations for how to improve the platform. Table 3 lists the modifications made to the website in response to these recommendations. Of note, not all feedback led to immediate changes to the website. For example, some participants requested a progress tracking feature that would enable them to quickly visualize which aspects of the program had already been completed. This change was not feasible immediately following usability testing; however, it has since been implemented in subsequent iterations of the website.

**Table 3.** Modifications made to the website in response to usability testing feedback.

Theme	Summary of issues identified by participants	Examples of website modifications
Aesthetics	Not visually engaging Images are generic Text hard to read when insufficiently contrasted with background	Added bright, colorful, young adult–relevant, session-consistent images throughout Ensured images reflected broad sociodemographic representation and ages Avoided business-like images Added colored text
Content	Relevance to cancer not obvious Purpose of both overall program and specific components (eg, interactive exercises) unclear Acronyms unknown	Added cancer-specific images throughout Added more detailed instructions throughout Spelled out acronyms
Function	Functionality of some aspects unclear (eg, interactive worksheets, achievements and events, favorites)	Develop standardized preprogram website orientation for participants to complete prior to being granted an account
Structure	Difficult to distinguish sections and topics	Adjusted font colors throughout to identify section headers, instructions, etc Added descriptive section titles Presented session text in multiple expandable sections

## Part 2: Examining the Feasibility and Acceptability of Delivering TOGETHER Through the Website

### Methods

#### Participants

Eligibility and recruitment for the feasibility trial mirrored that of usability testing, although feasibility trial participants were also required to have access to internet or cellular connectivity with sufficient bandwidth to participate in videoconferences. Participants who completed usability testing were permitted to enroll in the feasibility trial if desired. In such cases, participants provided informed consent for each stage of the study.

#### Procedures

The feasibility trial was preregistered on ClinicalTrials.gov (NCT05597228) [30] and consisted of a single instance of the 10-week TOGETHER group intervention. After providing informed consent, participants completed a 1-time website orientation videoconference meeting with a member of the research team. Participants were also mailed a physical copy of the TOGETHER participant workbook. The physical copies were provided because YAs who contributed to the development of the intervention content [17] recommended providing both physical and digital copies of intervention materials to program users.

Participants then completed a full administration of the facilitator-led TOGETHER group intervention delivered via the adapted website. All intervention sessions were held on a Health Insurance Portability and Accountability Act–compliant version of Zoom. The link to join each week’s session was visible on the website beginning 36 hours before the session was scheduled to start and remained visible until 2 hours after the session was scheduled to end. Sessions each lasted approximately 2 hours and occurred on the same day of the week at the same time for the duration of the program. Immediately following each session, participants in attendance were sent a unique link to complete a brief electronic survey assessing the acceptability of that session’s content and group dynamics. Participants completed a similar survey postintervention assessing the acceptability of the overall program. Participants completed an individual exit interview with a member of the research team postintervention to provide qualitative feedback on their experience. Participants also completed a battery of patient reported outcome measures at baseline and postintervention. The battery was consistent with the planned assessment protocol for a future, larger-scale trial; however, given the small sample size, we were not powered to detect effects and therefore did not analyze these data. Participants were compensated US $50 for completing the baseline and post-intervention assessment batteries, US $20 for completing the exit interview, and US $5 for each of the 10 weekly surveys completed. In total, feasibility trial participants had the opportunity to earn up to US $170.

#### TOGETHER Intervention

Details of the TOGETHER intervention have been previously published [17]. Briefly, each TOGETHER session consists of 3 main sections: learn and practice relaxation skills (first 30 min), practice skills derived from cognitive behavioral therapy principles (middle 60 min), and discuss YA-relevant health education topics (remaining 30 min). During each session, a facilitator guides participants through new content and leads interactive activities designed to reinforce the content and skills. Facilitators also create opportunities for participants to discuss their personal experiences and develop group rapport. Between sessions, participants complete home practice assignments to promote mastery of the intervention skills, and each session begins with a review of the prior week’s home practice. For this feasibility trial, sessions were facilitated by a predoctoral clinical psychology trainee (TKT) under the supervision of the study Principal Investigator (RSF). Sessions were video- and audio-recorded and reviewed during weekly supervision meetings to ensure intervention fidelity.

#### Primary Outcomes: Feasibility and Acceptability

Feasibility was measured by calculating study recruitment and retention rates and tracking session attendance [31]. Feasibility was defined as achieving a 50% recruitment rate, a 70% retention rate, and average attendance of ≥ 6 of the 10 sessions. These benchmarks were based on rates observed in past studies of similar behavioral interventions in diverse cancer survivors [19,32,33] and in accordance with our prior investigation of the feasibility of TOGETHER content [17]. Acceptability was measured with study-specific weekly and postintervention surveys our team previously developed to assess the acceptability of the TOGETHER content [17]. Survey items assessed participant satisfaction with multiple aspects of TOGETHER and were rated on a Likert scale ranging from 0 (strongly disagree) to 4 (strongly agree). For this study, a new item was added to the postintervention acceptability survey assessing the acceptability of the study website. The study was considered acceptable if average scores on the weekly and postintervention survey items were ≥2 (ie, neutral or better). Participants also completed the Satisfaction subscale of the USE questionnaire [27,28] postintervention and self-reported demographic and medical information at baseline.

#### Analysis

Descriptive statistics were used to summarize feasibility and acceptability metrics.

### Results

#### Participant Characteristics

Seven YA cancer survivors enrolled in the single-arm feasibility trial, 6 of whom had also participated in usability testing due to practicality reasons. Information about feasibility trial participants’ demographic and medical characteristics can be found in Table 1. On average, feasibility trial participants were 25 years old at the time of participation (range 18‐33) and were 22 years old at the time of incident cancer diagnosis (range 17‐32). The majority were female (n=5, 71%), non-Hispanic White (n=4, 57%), and had never been married (n=4, 57%). The most well represented cancer types included bone and soft tissue (n=2, 29%) and breast (n=2, 29%).

#### Feasibility

All feasibility metrics were met. Of 12 seemingly eligible YA cancer survivors who were approached for participation, 7 (58%) consented and enrolled in the study (Multimedia Appendix 2). All 7 participants attended the first group session, after which 1 withdrew, and 1 was lost to follow-up. Of the remaining 5 participants, all were retained through the postintervention assessment (71% of enrolled). Across all 7 participants, the average attendance was 7.1 of the 10 intervention sessions (SD 4.2); however, among the 5 participants who attended at least 2 sessions, the average attendance was 9.6 of the 10 sessions (SD 0.5).

#### Acceptability

All acceptability metrics were met. Tables4  5 show the average acceptability ratings for each of the 10 weekly sessions and for the overall program, respectively. Pooled average satisfaction with the individual weekly sessions was ≥3 for all items. Participants agreed to strongly agreed that they liked the sessions, the content was relevant and helpful, they felt confident with the content, and they felt comfortable and respected in the group. Similarly, at postintervention, the means for all items assessing overall program satisfaction were ≥3, with the exception of an item assessing satisfaction with the website. Based on feedback gathered in exit interviews, low ratings for satisfaction with the website were due to a timing feature that prevented participants from viewing data they had entered (eg, responses to home practice prompts) in subsequent weeks even though the data had been saved. This led some participants to express frustration with the website, particularly when reviewing the prior week’s home practice assignments at the start of each session. For example, when asked to share additional information about their experience in the program during exit interviews, one participant stated, “everything was great besides the homework assignments not saving” and another specified, “I would have liked using the website even more if it wouldn’t have erased the data. Besides that glitch it seemed quite self-explanatory and would’ve been used more.” This feature has since been fixed.

The Satisfaction subscale of the USE questionnaire supported the acceptability of the program website. The mean score on the overall subscale was 5.06 (SD 1.64), with individual item means ranging from 3.80 to 5.80. The item with the lowest average rating assessed if the website worked as desired, and no items received the lowest possible rating from any participant. When asked to identify the website’s most negative aspect(s), multiple participants identified the website not displaying past work properly. Conversely, when asked to identify the website’s most positive aspect(s), participants identified the “clean, friendly interface,” and described the website as “easy to navigate,” and “straight to the point.” Some participants also highlighted strengths of the overall program, including the exercises and home practice assignments as well as the value of meeting other YA cancer survivors and learning new tools and skills.

**Table 4.** Acceptability of TOGETHER’s 10 weekly sessions as delivered through the website. Possible scores range from 0 (strongly disagree) to 4 (strongly agree).

Item	Mean (SD)^a^	Observed range^b^
Overall, I liked this session.	3.30 (0.58)	2.44‐4.00
The content related to ______ was relevant to me.		
a) Relaxation	3.54 (0.55)	2.67‐4.00
b) Stress management	3.22 (0.77)	2.00‐4.00
c) Health topics	3.45 (0.57)	2.56‐4.00
The content related to ______ was helpful to me.		
a) Relaxation	3.29 (0.57)	2.67‐4.00
b) Stress management	3.25 (0.71)	2.00‐4.00
c) Health topics	3.19 (0.81)	2.00‐4.00
I feel confident with the new information and skills covered in this session.	3.37 (0.45)	3.00‐4.00
I felt comfortable expressing my experiences and feelings in the group.	3.46 (0.73)	2.00‐4.00
The other group members respected my experiences and feelings.	3.69 (0.41)	3.00‐4.00

a Means and standard deviations for each item were pooled across the 10 weekly surveys.

b Observed range of average values across the 10 weekly surveys.

**Table 5.** Acceptability of the overall TOGETHER program as delivered through the website. Possible scores range from 0 (strongly disagree) to 4 (strongly agree).

Item	Mean (SD)	Agreed or strongly agreed (%)
Overall, the content was relevant to me.	3.6 (0.5)	100
Overall, the content was helpful to me.	3.6 (0.9)	80
Overall, I liked the program content related to _____.		
a) Relaxation	3.4 (0.5)	100
b) Stress management	3.6 (0.5)	100
c) Health topics	3.2 (0.8)	80
I liked connecting with other YA^a^ cancer survivors in the weekly sessions.	4.0 (0.0)	100
I liked using the study website	2.8 (1.3)	60
I plan to continue using the skills I learned.	3.6 (0.5)	100
I would recommend the program to other YA cancer survivors.	3.8 (0.4)	100
Overall, I am glad I decided to participate.	3.8 (0.4)	100

a YA: young adult.

## Discussion

### Principal Findings

This manuscript describes the usability, feasibility, and acceptability of a website designed to host the TOGETHER group program for YA cancer survivors. We first adapted an existing digital platform to meet the needs of TOGETHER. Then, consistent with best practices for human-centered design [34], we conducted three waves of iterative usability testing to identify and address challenges with the website’s functionality, structure, content, and aesthetics as experienced by YA cancer survivors. Finally, we established the preliminary feasibility and acceptability of the TOGETHER program as delivered through the adapted website.

Usability testing identified desired changes to the website. Interestingly, many of these changes were consistent with feedback provided by YA cancer survivors who evaluated the “Roadmap to Parenthood” web-based decision tool for family building after cancer [35]. For example, usability testers of “Roadmap to Parenthood” reported that pages containing large amounts of content were overwhelming. Therefore, the designers divided content into separate pages and adjusted content to only be visible when a header was clicked. Font sizes and colors were also changed to better clarify the division of text. In Part 1 of this study, we made almost identical adjustments to the TOGETHER website. The consistency of these results suggests that individuals developing digital therapeutics for YA cancer survivors may benefit from considering these findings early in the prototype design process. Of note, usability testing for “Roadmap to Parenthood” also yielded findings related to visibility and navigation that did not emerge in the present study. However, an important distinction between TOGETHER and many other digital health interventions [36] is that TOGETHER is designed to be led in real-time by a group facilitator rather than self-guided. Consequently, users can be oriented to the website’s functionality prior to using it for the first time and the website does not need to stand alone, which may explain such discrepancies.

Although usability testing demonstrated that the TOGETHER website was easy to use and learn, the single-session nature of the usability testing precluded evaluation of time-based functionality of the website. This led to challenges during the feasibility trial. While the feasibility and acceptability of TOGETHER were generally strong, the website was the least acceptable aspect of the program. This low satisfaction was most likely due to participants being unable to view content they had previously input into the website at each group meeting. Contrary to participant understanding, the data had not been deleted; however, by the time each group meeting occurred, the prior week’s data were no longer displayed back for participants to view. This challenge impacted participant experiences of the website. By identifying this challenge at an early stage of testing, we have been able to adjust it prior to future, larger-scale testing of TOGETHER. We will re-evaluate the acceptability of the website following this change, consistent with the cyclic nature of user-centered design [37]. Despite this, the average reported acceptability of the website was still better than the identified threshold of 2.0, thus meeting this benchmark. Moreover, the overall acceptability of the website was comparable to what we found when testing the content as delivered via static, text-only PDF workbooks, further supporting the acceptability.

### Implications for Health Care and Research

The TOGETHER intervention is one of the first supportive care interventions for YA cancer survivors that fulfills their expressed desires for convenience (eg, digital delivery) and peer connection. By providing the program digitally, we have further increased its convenience, taking a step toward fulfilling YAs’ priorities and increasing dissemination potential. Future research is needed to test the intervention’s efficacy for improving HRQOL, to explore approaches for increasing intervention efficiency, and to explore strategies for implementation both within and outside of the healthcare system.

### Limitations

Usability testers were not given an opportunity to explore the website independently but rather were directed to complete prescribed tasks and answer specific questions. Although the prescribed tasks reflected what a YA would need to be able to do to engage with the intervention (eg, log in, navigate to session content), it is possible that additional opportunities to enhance usability could have been identified had the usability testers been given an opportunity for non-directed exploration. Of note, feasibility trial participants were given an opportunity to provide non-directed usability feedback during exit interviews. Another limitation is that the sample size for the feasibility trial was small, even when combined with our prior testing of the TOGETHER content. This is particularly true given that, due to practicality, 6 participants were included in both the usability testing sample and the feasibility trial sample, which could have impacted our findings. Further data are needed to confirm the observed results. The small sample size also precluded evaluation of the intervention’s effects on theorized outcomes.

### Conclusions

Study results support the usability, feasibility, and acceptability of the TOGETHER program and website. The incorporation of YA cancer survivors’ feedback into the development of the intervention content and delivery platform is consistent with their expressed desires to be actively engaged in research [21] and likely contributed to the high observed acceptability. Additionally, by enabling digital delivery of TOGETHER, we have directly responded to YAs’ expressed priority that interventions be convenient [20-22]. Larger-scale testing is needed to establish the efficacy of TOGETHER and explore alternative study designs to increase efficiency.

## Supplementary material

10.2196/58014 Multimedia Appendix 1 TOGETHER session 1 screenshot.

10.2196/58014 Multimedia Appendix 2 Participant flow diagram.

## Abbreviations

HRQOL: health-related quality of life
USE: Usefulness, Satisfaction, and Ease of Use questionnaire
YA: young adult

## References

[1] (2020). Cancer facts and figures 2020: special section: cancer in adolescents and young adults. American Cancer Society.

[2] Patterson P, McDonald FEJ, Zebrack B, Medlow S (2015). Emerging issues among adolescent and young adult cancer survivors. Semin Oncol Nurs.

[3] Parsons HM, Harlan LC, Lynch CF (2012). Impact of cancer on work and education among adolescent and young adult cancer survivors. J Clin Oncol.

[4] Kwak M, Zebrack BJ, Meeske KA (2013). Trajectories of psychological distress in adolescent and young adult patients with cancer: a 1-year longitudinal study. J Clin Oncol.

[5] D’Agostino NM, Edelstein K (2013). Psychosocial challenges and resource needs of young adult cancer survivors: implications for program development. J Psychosoc Oncol.

[6] Quinn GP, Gonçalves V, Sehovic I, Bowman ML, Reed DR (2015). Quality of life in adolescent and young adult cancer patients: a systematic review of the literature. Patient Relat Outcome Meas.

[7] Lang MJ, David V, Giese-Davis J (2015). The age conundrum: a scoping review of younger age or adolescent and young adult as a risk factor for clinical distress, depression, or anxiety in cancer. J Adolesc Young Adult Oncol.

[8] De R, Zabih V, Kurdyak P (2020). Psychiatric disorders in adolescent and young adult-onset cancer survivors: a systematic review and meta-analysis. J Adolesc Young Adult Oncol.

[9] Kwak M, Zebrack BJ, Meeske KA (2013). Prevalence and predictors of post-traumatic stress symptoms in adolescent and young adult cancer survivors: a 1-year follow-up study. Psychooncology.

[10] Sanford SD, Zhao F, Salsman JM, Chang VT, Wagner LI, Fisch MJ (2014). Symptom burden among young adults with breast or colorectal cancer. Cancer.

[11] DeRouen MC, Smith AW, Tao L (2015). Cancer-related information needs and cancer’s impact on control over life influence health-related quality of life among adolescents and young adults with cancer. Psychooncology.

[12] Kent EE, Smith AW, Keegan THM (2013). Talking about cancer and meeting peer survivors: social information needs of adolescents and young adults diagnosed with cancer. J Adolesc Young Adult Oncol.

[13] Bellver-Pérez A, Peris-Juan C, Santaballa-Beltrán A (2019). Effectiveness of therapy group in women with localized breast cancer. Int J Clin Health Psychol.

[14] Schellekens MPJ, Tamagawa R, Labelle LE (2017). Mindfulness-Based Cancer Recovery (MBCR) versus Supportive Expressive Group Therapy (SET) for distressed breast cancer survivors: evaluating mindfulness and social support as mediators. J Behav Med.

[15] Duncan M, Moschopoulou E, Herrington E (2017). Review of systematic reviews of non-pharmacological interventions to improve quality of life in cancer survivors. BMJ Open.

[16] Rehse B, Pukrop R (2003). Effects of psychosocial interventions on quality of life in adult cancer patients: meta analysis of 37 published controlled outcome studies. Patient Educ Couns.

[17] Oswald LB, Lyleroehr M, Gudenkauf LM (2022). Development and initial testing of TOGETHER-YA: an eHealth-delivered and group-based psychosocial intervention for young adult cancer survivors. Support Care Cancer.

[18] Antoni MH (2003). Stress management effects on psychological, endocrinological, and immune functioning in men with HIV infection: empirical support for a psychoneuroimmunological model. Stress.

[19] Penedo FJ, Fox RS, Oswald LB (2020). Technology-based psychosocial intervention to improve quality of life and reduce symptom burden in men with advanced prostate cancer: results from a randomized controlled trial. Int J Behav Med.

[20] Rabin C, Simpson N, Morrow K, Pinto B (2013). Intervention format and delivery preferences among young adult cancer survivors. Int J Behav Med.

[21] Benedict C, Victorson D, Love B (2018). The audacity of engagement: hearing directly from young adults with cancer on their attitudes and perceptions of cancer survivorship and cancer survivorship research. J Adolesc Young Adult Oncol.

[22] Oswald LB, Victorson DE, Fox RS (2021). Young adult cancer survivors’ preferences for supportive interventions. Psychooncology.

[23] Kinzie MB, Cohn WF, Julian MF, Knaus WA (2002). A user-centered model for web site design: needs assessment, user interface design, and rapid prototyping. J Am Med Inform Assoc.

[24] Puccinelli M, Seay J, Otto A (2022). An adapted cognitive behavioral stress and self-management intervention for sexual minority men living with HIV and cancer using the SmartManage eHealth platform: protocol and study design. JMIR Res Protoc.

[25] Sauro J, Lewis JR (2016). Quantifying the User Experience: Practical Statistics for User Research.

[26] Graham AK, Lattie EG, Mohr DC (2019). Experimental therapeutics for digital mental health. JAMA Psychiatry.

[27] Lund AM (2001). Measuring usability with the use questionnaire. Usability Interface.

[28] Gao M, Kortum P, Oswald F (2018). Psychometric evaluation of the USE (Usefulness, Satisfaction, and Ease of USE) Questionnaire for reliability and validity. Proc Hum Factors Ergon Soc Annu Meet.

[29] Gale RC, Wu J, Erhardt T (2019). Comparison of rapid vs in-depth qualitative analytic methods from a process evaluation of academic detailing in the Veterans Health Administration. Implement Sci.

[30] University of Arizona (2024). Tech-TYA: eHealth platform to deliver group intervention for YA cancer survivors. ClinicalTrials.gov.

[31] Bowen DJ, Kreuter M, Spring B (2009). How we design feasibility studies. Am J Prev Med.

[32] Yanez B, McGinty HL, Mohr DC (2015). Feasibility, acceptability, and preliminary efficacy of a technology-assisted psychosocial intervention for racially diverse men with advanced prostate cancer. Cancer.

[33] Antoni MH, Lechner SC, Kazi A (2006). How stress management improves quality of life after treatment for breast cancer. J Consult Clin Psychol.

[34] Lyon AR, Koerner K (2016). User-centered design for psychosocial intervention development and implementation. Clin Psychol (New York).

[35] Benedict C, Dauber-Decker KL, Ford JS (2022). Development of a web-based decision aid and planning tool for family building after cancer (roadmap to parenthood): usability testing. JMIR Cancer.

[36] Devine KA, Viola AS, Coups EJ, Wu YP (2018). Digital health interventions for adolescent and young adult cancer survivors. JCO Clin Cancer Inform.

[37] Mohr DC, Lyon AR, Lattie EG, Reddy M, Schueller SM (2017). Accelerating digital mental health research from early design and creation to successful implementation and sustainment. J Med Internet Res.

