# Supplementary figures and images for: Delivering a Group-Based Quality of Life Intervention to Young Adult Cancer Survivors via a Web Platform: Feasibility Trial
Source: JMIR Cancer. 2024 Dec 4;10:e58014. doi: 10.2196/58014 (PMC11634045; doi:10.2196/58014)

## Multimedia Appendix 1

Multimedia Appendix 1. TOGETHER Session 1 screenshot


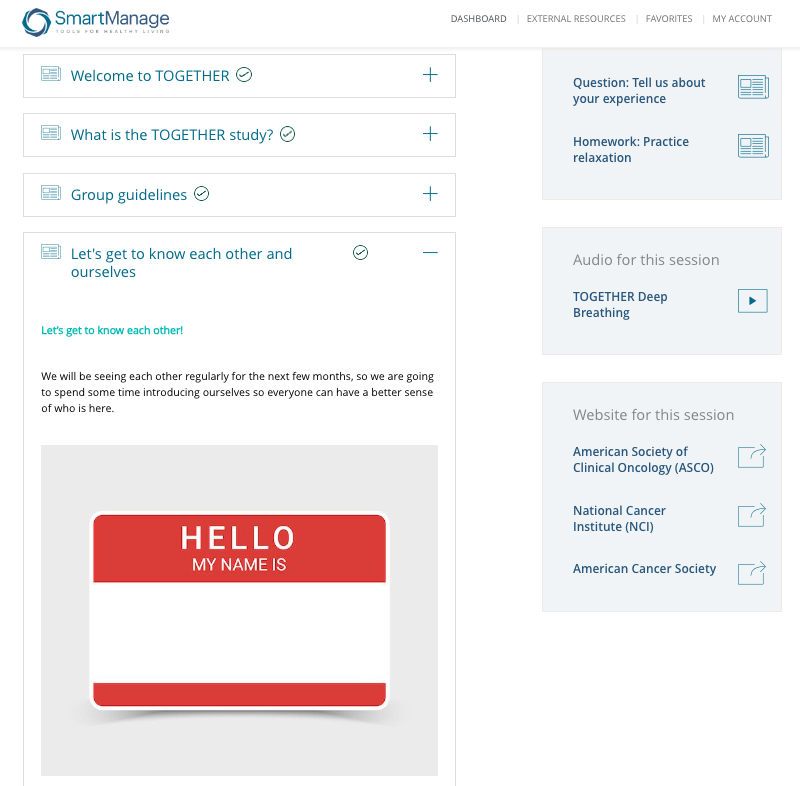

Supplement: Multimedia Appendix 1 [file cancer-v10-e58014-s001.docx]

## Multimedia Appendix 2

Participant flow diagram


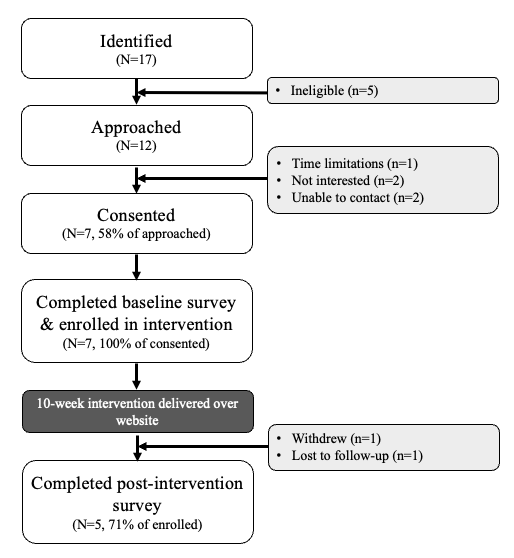

Supplement: Multimedia Appendix 2 [file cancer-v10-e58014-s002.docx]
